# The effects of icariin on the expression of HIF-1α, HSP-60 and HSP-70 in PC12 cells suffered from oxygen–glucose deprivation-induced injury

**DOI:** 10.1080/13880209.2017.1281968

**Published:** 2017-01-31

**Authors:** Zhen-Tao Mo, Wen-Na Li, Yu-Rong Zhai, Shu-Ying Gao

**Affiliations:** Department of Pharmacology of Zhuhai Campus, Zunyi Medical University, Zhuhai, Guangdong, China

**Keywords:** Heat shock protein, neuron-specific enolase, cell viability

## Abstract

**Context:** The effects of icariin, a chief constituent of ﬂavonoids from *Epimedium brevicornum* Maxim (Berberidaceae), on the levels of HIF-1α, HSP-60 and HSP-70 remain unknown.

**Objective:** To explore the effects of icariin on the levels of HSP-60, HIF-1α and HSP-70 neuron-specific enolase (NSE) and cell viability.

**Materials and methods:** PC12 cells were treated with icariin (10^−7^, 10^−6^ or 10^−5 ^mol/L) for 3 h (1 h before oxygen–glucose deprivation (OGD) plus 2 h OGD). HSP-60, HIF-1α, HSP-70 and NSE were measured using enzyme-linked immunosorbent assay (ELISA). Cell viability was determined by metabolic 3-(4,5-dimethylthiazol-2-yl)-2,5-diphenyltetrazolium bromide (MTT) assay.

**Results:** After 2 h OGD, levels of HIF-1α, HSP-60, HSP-70 and NSE were increased significantly (HIF-1α: 33.3 ± 1.9 ng/L, HSP-60: 199 ± 16 ng/L, HSP-70: 195 ± 17 ng/L, NSE: 1487 ± 125 ng/L), and cell viability was significantly decreased (0.26 ± 0.03), while icariin (10^−7^, 10^−6^, or 10^−5 ^mol/L) significantly reduced the contents of HIF-1α, HSP-60, HSP-70 and NSE (HIF-1α: 14.1 ± 1.4, 22.6 ± 1.8, 15.7 ± 2.1, HSP-60: 100 ± 12, 89 ± 6, 113 ± 11, HSP-70: 139 ± 9, 118 ± 7, 95 ± 9 and NSE: 1121 ± 80, 1019 ± 52, 731 ± 88), and improved cell viability (0.36 ± 0.03, 0.38 ± 0.04, 0.37 ± 0.03) in OGD-treated PC12 cells.

**Discussion and conclusion:** These results indicate that the protective mechanisms of icariin against OGD-induced injury may be related to down-regulating the expression of HIF-1α, HSP-60 and HSP-70.

## Introduction

Icariin is a major bioactive component extracted from *Epimedium brevicornum* Maxim (Berberidaceae) (Li et al. 2015). It has shown various neuroprotective effects on ischaemic stroke. It can improve neurological scores, and reduce cerebral infarct size and brain oedema (Zhu et al. 2010a). Its neuroprotective mechanisms include protecting mitochondria and reducing oxidative stress, calcium overload and apoptosis (Zhu et al. 2010a; Li et al. 2005). PC12 cell line is derived from a pheochromocytoma of the rat adrenal medulla. It is widely used as an *in vitro* model of cerebral ischaemia (Zhu et al. 2010b; Mo et al. 2012). Our previous study found that icariin could reduce the contents of tumour necrosis factor (TNF)-α, interleukin (IL)-1β and IL-6 in the supernatant fluid of PC12 cells injured by oxygen–glucose deprivation **(**OGD) (Mo et al. 2015). It is reported that hypoxia inducible factor (HIF)-1α, extracellular heat shock protein (HSP)-60 and extracellular HSP-70 could stimulate the synthesis and release of these inflammatory cytokines (Tannahill et al. 2013; Tian et al. 2013; Dvoriantchikova et al. 2014). Extracellular HSP-60 could increase HIF-1α expression, an activity subunit of HIF, which could stimulate HSP-70 expression (Ban et al. 2010; Tsuchida et al. 2014). Whether icariin has effects on the expression of HIF-1α, HSP-60 and HSP-70, the upstream signalling inflammatory cytokines, is unknown. Therefore, the levels of HIF-1α, HSP-60 and HSP-70, neuron-specific enolase (NSE), a marker reflecting neuronal damage, and cell viability were measured in this study to explore the neuroprotective mechanisms of icariin against OGD-induced injury.

## Materials and methods

### Icariin preparation

Icariin (purity 98.3% by HPLC) was purchased from Nanjing Zelang Pharmaceutical Technology Co., Ltd (Nanjing, China). Icariin was dissolved in DMSO to 10^−3 ^mol/L and stored at −20 °C. When icariin was used, 10^−3 ^mol/L icariin was diluted with Earle’s balanced salt solution or full culture medium to the final concentration of 10^−7^, 10^−6^ and 10^−5 ^mol/L. Earle’s balanced salt solution was composed of 116 mmol/L NaCl, 5.4 mmol/L KCl, 0.8 mmol/L MgSO_4_, 1 mmol/L NaH_2_PO_4_, 0.9 mmol/L CaCl_2_ and 10 mg/L phenol red. The full culture medium contained 90% 4.5 g/mL glucose Dulbecco’s modified Eagle’s medium (DMEM) (Gibco, NY), 5% heat-inactivated foetal bovine serum(Gibco, NY) and 5% horse serum (Gibco, NY).

### Cell cultures

PC12 cells (donated by Dr. G.Z. Cui, University of Macau) were seeded in 25 cm^2^ polystyrene flasks (Corning Costar Corp, NY) with full culture medium as described above. The cells were incubated under an atmosphere of 95% air and 5% CO_2_. Culture medium was replaced every 48 h.

### Oxygen–glucose deprivation (OGD)

PC12 cells were deprived of oxygen and glucose for 2 h to simulate ischaemic injury *in vitro* as previously described (Mo et al. 2012). PC12 cells were washed with phosphate buffer solution (PBS) for one time and incubated in Earle’s balanced salt solution as described above. Then, the cells were incubated in a hypoxia chamber (HF100, Heal Force, China) with a compact gas oxygen controller to maintain oxygen concentration at 1% by injecting a gas mixture of 94% N_2_ and 5% CO_2_ for 2 h. Normal control cells were incubated in a regular cell culture incubator (HF90, Heal Force, China) under normoxic conditions.

### Drug administration

PC12 cells were incubated with full culture medium containing icariin (10^−7^, 10^−6^ or 10^−5 ^mol/L) or nimodipine (10 μmol/L) under normoxic conditions for 1 h before hypoxia. The full culture medium containing drug was discarded. The cells were rinsed once with PBS, and incubated with Earle’s balanced salt solution containing icariin (10^−7^, 10^−6^, or 10^−5 ^mol/L) or nimodipine (10 μmol/L) for 2 h of hypoxia.

### Metabolic 3-(4,5-dimethylthiazol-2-yl)-2,5-diphenyltetrazolium bromide (MTT) assay

PC12 cells were seeded into 96-well plastic plates with 0.1 mL at the density of 1 × 10^5^ cells/mL. It contained 10 samples in normal control group, OGD-treated group and OGD plus drug-treated groups, respectively. Then, after growth for 48 h, the cells were treated with OGD and drug administration as described above. After OGD and drug administration, the MTT colorimetric assay was performed. 10 μL of MTT (10 mg/mL) (Sigma, CA) was added to each well. Plates were incubated at 37 °C in normoxia for 4 h. Dimethyl sulfoxide (DMSO, Sangon Biotech, Shanghai, China) (150 μL) was added to each well for 10 min to dissolve the dark blue crystals and the absorbance was read at 570 nm on a microplate reader (Multiskan Mk3, Thermo Scientific, MA).

### Enzyme-linked immunosorbent assay (ELISA)

PC12 cells were seeded into 6-well plastic plates with 1 mL at the density of 5 × 10^5^ cells/mL. It contained six samples in normal control group, OGD-treated group and OGD plus drug-treated groups, respectively. Then, after growth for 48 h, the cells were treated with OGD and drug administration as described above. After OGD and drug administration, 100 μL per well of cell culture supernatant fluid was used for measuring HSP-60 (Guangzhou Biolink Biotechnology Co, Ltd, China), HIF-1α (Guangzhou Biolink Biotechnology Co, Ltd, China), HSP-70 (Guangzhou Biolink Biotechnology Co, Ltd, China) and NSE (Guangzhou Biolink Biotechnology Co, Ltd, China) using ELISA according to the corresponding manufacturer’s instructions.

### Statistical analysis

Measurement data were expressed as mean ± standard deviation (Mean ± SD). Statistical significance was determined by two independent samples *t* test. A value of *p* < 0.05 was considered significant. All statistical analyses were performed with version SPSS 19.0 statistical software.

## Results

### Effect of icariin on cell viability in OGD-treated PC12 cells

**Figure 1.**
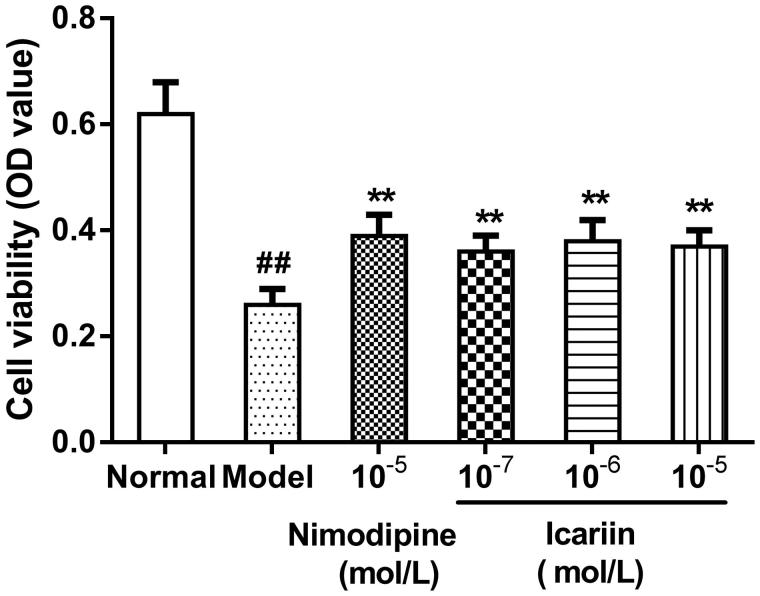
Effect of icariin on cell viability in OGD-treated PC12 cells. Model control cells were treated with 2 h OGD. The treated cells were incubated with icariin (10^−7^, 10^−6^ or 10^−5^ mol/L) or nimodipine (10 μmol/L) 1 h before OGD and 2 h throughout OGD. Normal control cells were incubated in a regular cell culture incubator under normoxic conditions. After these treatments, cell viability was analyzed using MTT assay. Mean ± SD for 10 samples. ##*p* < 0.01 vs normal control group. **p < 0.01 vs model control group.

Cell viability [optical density value (OD value)] was 0.62 ± 0.06 in normal control cells. It was significantly decreased in OGD-treated cells (0.26 ± 0.03, *p* < 0.01). After treatment with icariin (10^−7^, 10^−6^ and 10^−5 ^mol/L) or nimodipine (10 μmol/L), respectively, cell viability was dramatically increased (*p* < 0.01) (Figure 1).

### Effect of icariin on HIF-1α levels in OGD-treated PC12 cells

**Figure 2.**
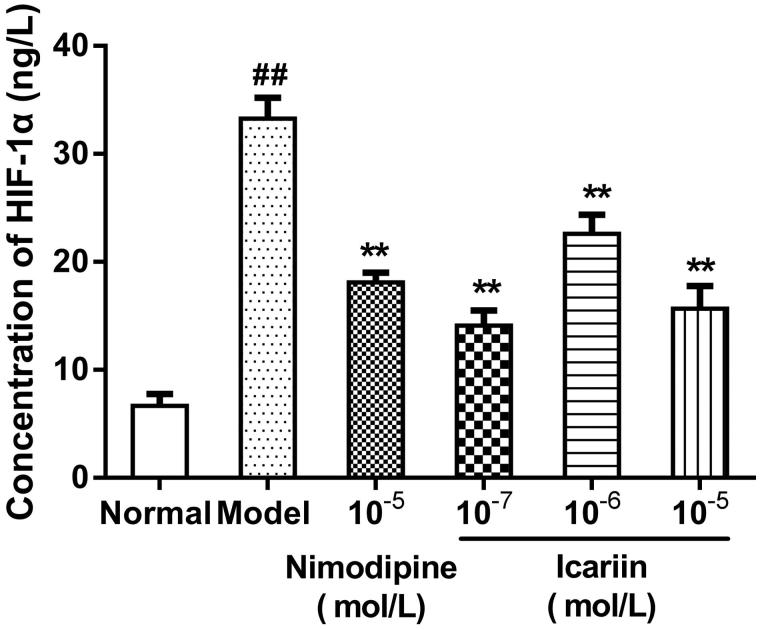
Effect of icariin on HIF-1α levels in OGD-treated PC12 cells. Model control cells were treated with 2 h OGD. The treated cells were incubated with icariin (10^−7^, 10^−6^ or 10^−5^ mol/L) or nimodipine (10 μmol/L) 1 h before OGD and 2 h throughout OGD. Normal control cells were incubated in a regular cell culture incubator under normoxic conditions. After these treatments, HIF-1α levels in cell supernatant fluid were analyzed using ELISA assay. Mean ± SD for six samples. ##*p* < 0.01 vs normal control group. **p < 0.01 vs model control group.

In OGD-treated cells, HIF-1α levels (33.3 ± 1.9 ng/L) were increased to fivefold compared with normal control cells (6.7 ± 1.1 ng/L, *p* < 0.01). Icariin treatment at concentrations range from 10^−7^ to 10^−5 ^mol/L reduced HIF-1α levels significantly, compared to OGD-treated cells (*p* < 0.01) (Figure 2).

### Effect of icariin on HSP-60 and HSP-70 levels in OGD-treated PC12 cells

**Figure 3.**
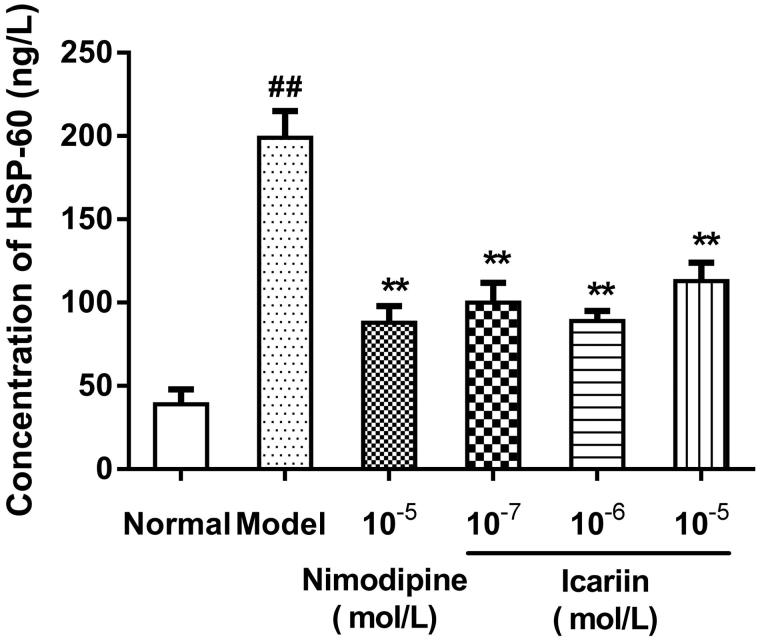
Effect of icariin on HSP-60 levels in OGD-treated PC12 cells. Model control cells were treated with 2 h OGD. The treated cells were incubated with icariin (10^−7^, 10^−6^ or 10^−5^ mol/L) or nimodipine (10 μmol/L) 1 h before OGD and 2 h throughout OGD. Normal control cells were incubated in a regular cell culture incubator under normoxic conditions. After these treatments, HSP60 levels in cell supernatant fluid were analyzed using ELISA assay. Mean ± SD for six samples. ##*p* < 0.01 vs normal control group. **p < 0.01 vs model control group.

**Figure 4.**
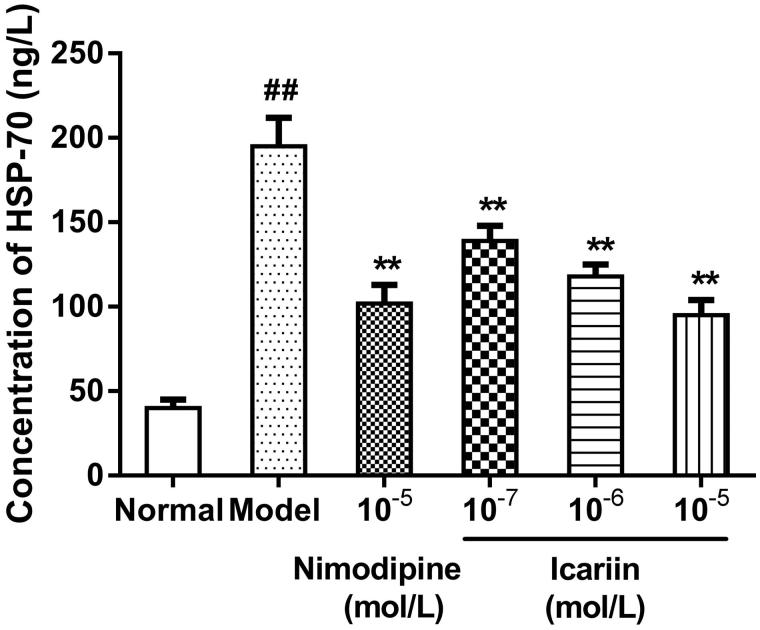
Effect of icariin on HSP-70 levels in OGD-treated PC12 cells. Model control cells were treated with 2 h OGD. The treated cells were incubated with icariin(10^−7^, 10^−6^ or 10^−5^ mol/L) or nimodipine (10 μmol/L) 1 h before OGD and 2 h throughout OGD. Normal control cells were incubated in a regular cell culture incubator under normoxic conditions. After these treatments, HSP70 levels in cell supernatant fluid were analyzed using ELISA assay. Mean ± SD for six samples. ##*p* < 0.01 vs normal control group. **p < 0.01 vs model control group.

In OGD-treated cells, HSP-60 and HSP-70 levels were increased to 5.1- and 4.9-fold, respectively, (199 ± 16 ng/L in HSP-60, 195 ± 17 ng/L in HSP-70, respectively), compared to normal control cells (39 ± 9 ng/L in HSP-60, 40 ± 5 ng/L in HSP-70, respectively). Icariin treatment at concentrations range from 10^−7^ to 10^−5 ^mol/L reduced HSP-60 and HSP-70 levels significantly, compared to OGD-treated cells (*p* < 0.01) (Figures 3, 4).

### Effect of icariin on NSE levels in OGD-treated PC12 cells

**Figure 5.**
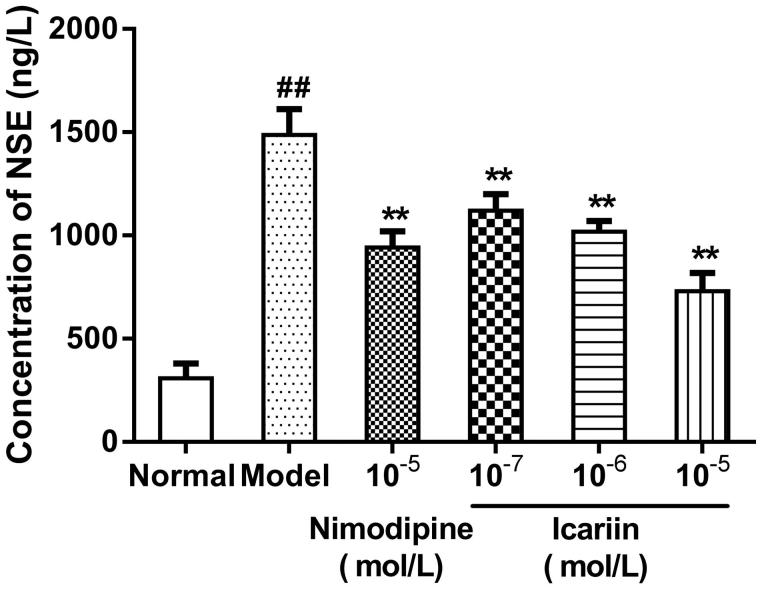
Effect of icariin on NSE levels in OGD-treated PC12 cells. Model control cells were treated with 2 h OGD. The treated cells were incubated with icariin(10^−7^, 10^−6^ or 10^−5^ mol/L) or nimodipine (10 μmol/L) 1 h before OGD and 2 h throughout OGD. Normal control cells were incubated in a regular cell culture incubator under normoxic conditions. After these treatments, NSE levels in cell supernatant fluid was analyzed using ELISA assay. Mean ± SD for six samples. ##*p* < 0.01 vs normal control group. **p < 0.01 vs model control group.

In OGD-treated cells, NSE levels were increased to 4.8-fold (1487 ± 125 ng/L), compared to normal control cells (309 ± 72 ng/L). Icariin treatment at concentrations range from 10^−7^ to 10^−5 ^mol/L reduced NSE levels significantly, compared to OGD-treated cells (*p* < 0.01) (Figure 5).

## Discussion

Neuroprotective mechanisms of icariin include reducing oxidative stress, inhibiting proinflammatory role of microglia, regulating metabolism of monoamine neurotransmitter, increasing synthesis and release of neurotrophic factors and promoting angiogenesis (Chung et al. 2008; He et al. 2010; Li et al. 2005, 2010; Zeng et al. 2010). Our previous study showed that icariin reduced the contents of TNF-α, IL-1β and IL-6 in OGD-treated PC12 cells, and play a protective role in the injured PC12 cells. It is reported that HIF-1α, extracellular HSP-60 and extracellular HSP-70 could stimulate the synthesis and release of these inflammatory cytokines. Therefore, in the present study, we evaluated whether icariin reduced the release of NSE and increased cell viability through decreasing HIF-1α, HSP-60 and HSP-70 levels in the supernatants of OGD-treated PC12 cells.

HIF is a heterodimeric cytokine composed of α subunit which affects the content and activity of HIF and β-subunit. Under normoxic conditions, only HIF-1β exists, because HIF-1α is not stable, and is degraded by ubiquitin-proteasome system. Under hypoxic conditions, ubiquitin-proteasome system is inhibited, and HIF-1α is stabilized, accumulated and transfers from the cytoplasm to the nucleus in combination with HIF-1β to form active HIF-1 which binds to the hypoxia response elements of genes, and stimulates the transcription of these target genes, including glucose transporter-1, the glycolytic enzymes and vascular endothelial growth factor (VEGF), responsible for cellular survival under hypoxic conditions (Willam et al. 2006; Jung et al. 2014). However, overexpression of HIF promotes glycolysis providing energy for cells, but also leads to the accumulation of lactic acid, resulting in the increase of lactic acidosis and necrosis (Blum et al. 2005). HIF can also activate inflammatory responses through enhancing several proinflammatory cytokines levels, such as TNF-α, IL-1β, cyclooxygenase-2 and inducible nitric oxide synthase (Feinman et al. 2010; Tannahill et al. 2013). It is reported that HIF-1α levels measured using ELISA were increased after hypoxia-ischemia (Chu & Jones 2016). In the present study, we also showed that HIF-1α levels were increased in OGD-injured PC12 cells, and icariin decreased HIF-1α levels in these model cells (Figure 2).

Heat-shock proteins (HSPs) are a family of highly conserved proteins during biological evolution. HSPs exist in all living organisms, from bacteria to humans (Giffard et al. 2008). They were up-regulated rapidly in response to harmful stimuli, such as ischemia, trauma and oxidative stress, and helped to fold proteins correctly, and helped to degrade and remove the denatured proteins (Zhang et al. 2012; Kacimi & Yenari 2015; O'Neill et al. 2015). HSP-60 and HSP-70 play an important role in cerebral ischemia. HSP-60 is a sensitive indicator of mitochondrial damage, and HSP-70 is a sensitive indicator of cell injured by hypoxia (Chandra et al. 2007; Awad et al. 2008). Extracellular HSP-60 released by the cells subjected to ischemia can induce inflammation through activating and up-regulating toll-like receptors (TLRs) (Tian et al. 2013). HSP-60 can also cause HIF-1α accumulation (Ban et al. 2010). Many studies has demonstrated that HSP-70 has protective effects on cerebral ischaemia, but over-expression of HSP-70 promotes TNF-mediated apoptosis (Ran et al. 2004; van der Weerd et al. 2005; Shevtsov et al. 2014). Down-regulation of HSP-70 reduces apoptosis (Tatsuta et al. 2014). Extracellular HSP-70 activates TLRs in the cell surface, and promotes an inflammatory response which exacerbates ischaemia injury (Asea et al. 2002; Mathur et al. 2011; Dvoriantchikova et al. 2014). HIF-1α also induces HSP-70 expression (Tsuchida et al. 2014).

NSE is a sensitive indicator of the degree of brain damage (Yoshinori et al. 1995). It is specifically found in the nervous system of mature nerve cells and nerve endocrine cells. In normal conditions, the concentration of NSE in the peripheral blood is very low due to the integrity of the brain cells and blood–brain barrier. When the brain is injured, NSE transfers from the injured brain to the peripheral blood, so NSE levels in the peripheral blood can reflect the degree of brain damage (Dauberschmidt et al. 1983; Yoshinori et al. 1995). NSE is also released to the cell culture medium in injured PC12 cells (Lin et al. 2008; Xue et al. 2013). So, NSE is also an indicator of PC12 cells damage.

Our previous study has indicated that nimodipine, a calcium antagonist, can reduce intracellular free calcium concentration and increased cell viability in OGD/R-treated PC12 cells (Mo et al. 2012). It is reported that nimodipine decreased ROS concentrations and HIF-1α expression in hippocampal neurons exposed to ketamine (Yan et al. 2014). Therefore, this work set nimodipine as a positive control group. In the present study, we showed that icariin decreased the expression of HSP-70 in a dose-dependent manner, increased the cell viability, and decreased the expression of HSP-60 and HIF-1α in a dose-independent manner at the concentrations of 10^−7^, 10^−6^ and 10^−5 ^mol/L. The expression of the HSP-60, HIF-1α and HSP-70 was not coincident among different concentrations of icariin, which did not mean either higher or lower concentration generate a stronger effect. Icariin is not an agonist or inhibitor of HSP-60, so the experimental data did not reflects that when concentration of icariin and HSP-60 expression is high, HIF-1α expression is high, or when HSP-60 expression is low, HIF-1α expression is low. HIF-1α, extracellular HSP-60 and extracellular HSP-70 can stimulate inflammatory responses, which aggravate ischaemia injury. The present study demonstrated that icariin reduced HIF-1α, HSP-60, HSP-70 and NSE levels, and increased cell viability in OGD-injured PC12 cells. Our previous study showed that icariin decreased TNF-α, IL-1β and IL-6 levels in PC12 cells following OGD injury. Taken together, these results and the above listed studies suggest that icariin may attenuate OGD-induced injury in PC12 cells through inhibiting the expression of HIF-1α, HSP-60 and HSP-70, and subsequently reducing the levels of inflammatory cytokines.
